# Hair Cortisol in Twins: Heritability and Genetic Overlap with Psychological Variables and Stress-System Genes

**DOI:** 10.1038/s41598-017-11852-3

**Published:** 2017-11-10

**Authors:** Liz Rietschel, Fabian Streit, Gu Zhu, Kerrie McAloney, Josef Frank, Baptiste Couvy-Duchesne, Stephanie H. Witt, Tina M. Binz, CORtisolNETwork (CORNET) Consortium, Jennifer L. Bolton, Caroline Hayward, Nese Direk, Anna Anderson, Jennifer Huffman, James F. Wilson, Harry Campbell, Igor Rudan, Alan Wright, Nicholas Hastie, Sarah H. Wild, Fleur P. Velders, Albert Hofman, Andre G. Uitterlinden, Jari Lahti, Katri Räikkönen, Eero Kajantie, Elisabeth Widen, Aarno Palotie, Johan G. Eriksson, Marika Kaakinen, Marjo-Riitta Järvelin, Nicholas J. Timpson, George Davey Smith, Susan M. Ring, David M. Evans, Beate St Pourcain, Toshiko Tanaka, Yuri Milaneschi, Stefania Bandinelli, Luigi Ferrucci, Pim van der Harst, Judith GM Rosmalen, Stephen JL Bakker, Niek Verweij, Robin PF Dullaart, Anubha Mahajan, Cecilia M. Lindgren, Andrew Morris, Lars Lind, Erik Ingelsson, Laura N. Anderson, Craig E. Pennell, Stephen J. Lye, Stephen G. Matthews, Joel Eriksson, Dan Mellstrom, Claes Ohlsson, Jackie F. Price, Mark WJ Strachan, Rebecca M. Reynolds, Henning Tiemeier, Major Depressive Disorder Working Group of the Psychiatric Genomics Consortium (PGC), Stephan Ripke, Manuel Mattheisen, Abdel Abdellaoui, Mark J. Adams, Esben Agerbo, Tracy M. Air, Till FM Andlauer, Silviu-Alin Bacanu, Marie Bækvad-Hansen, Aartjan TF Beekman, David A. Bennett, Klaus Berger, Tim B. Bigdeli, Jonas Bybjerg-Grauholm, Enda M. Byrne, Na Cai, Enrique Castelao, Toni-Kim Clarke, Jonathan RI Coleman, Converge Consortium, Nick Craddock, Udo Dannlowski, Gareth Davies, Gail Davies, Eco. J. C. de Geus, Philip De Jager, Ian J. Deary, Franziska Degenhardt, Erin C. Dunn, Erik A. Ehli, Thalia C. Eley, Valentina Escott-Price, Tõnu Esko, Hilary K. Finucane, Michael Gill, Scott D. Gordon, Jakob Grove, Lynsey S. Hall, Thomas F. Hansen, Christine Søholm Hansen, Thomas F. Hansen, Andrew C. Heath, Anjali K. Henders, Stefan Herms, Per Hoffmann, Georg Homuth, Carsten Horn, Jouke- Jan Hottenga, David Hougaard, Hailiang Huang, Marcus Ising, Rick Jansen, Eric Jorgenson, Stefan Kloiber, James A Knowles, Warren W. Kretzschmar, Jesper Krogh, Zoltán Kutalik, Maren Lang, Glyn Lewis, Yihan Li, Donald J. MacIntyre, Pamela AF Madden, Jonathan Marchine, Hamdi Mbarek, Peter McGuffin, Divya Mehta, Andres Metspalu, Christel M. Middeldorp, Evelin Mihailov, Lili Milani, Grant W. Montgomery, Sara Mostafavi, Niamh Mullins, Matthias Nauck, Bernard Ng, Merete Nordentoft, Dale R. Nyholt, Michael C. O’Donovan, Paul F. O’Reilly, Hogni Oskarsson, Michael J. Owen, Sara A. Paciga, Carsten Bøcker Pedersen, Marianne Giørtz Pedersen, Nancy L. Pedersen, Michele L. Pergadia, Roseann E. Peterson, Erik Pettersson, Wouter J. Peyrot, David J. Porteous, Danielle Posthuma, James B. Potash, Jorge A. Quiroz, John P. Rice, Brien P. Riley, Margarita Rivera, Douglas M. Ruderfer, Saira Saeed Mirza, Robert Schoevers, Ling Shen, Jianxin Shi, Engilbert Sigurdsson, Grant CB Sinnamon, Johannes H. Smit, Daniel J. Smith, Jordan W. Smoller, Hreinn Stephansson, Stacy Steinberg, Jana Strohmaier, Katherine E. Tansey, Alexander Teumer, Wesley Thompson, Pippa A. Thomson, Thorgeir E. Thorgeirsson, Jens Treutlein, Maciej Trzaskowski, Daniel Umbricht, Sandra van der Auwera, Gerard van Grootheest, Albert M. van Hemert, Alexander Viktorin, Henry Völzke, Yunpeng Wang, Bradley T. Webb, Myrna M. Weissman, Jürgen Wellmann, Gonneke Willemsen, Hualin S. Xi, Bernhard T. Baune, Douglas H. R. Blackwood, Dorret I. Boomsma, Anders D. Børglum, Henriette N. Buttenschøn, Sven Cichon, Enrico Domenici, Jonathan Flint, Hans J. Grabe, Steven P. Hamilton, Kenneth S. Kendler, Qingqin S. Li, Susanne Lucae, Patrik K. Magnusson, Andrew M. McIntosh, Ole Mors, Preben Bo Mortensen, Bertram Müller-Myhsok, Brenda WJH Penninx, Roy H. Perlis, Martin Preisig, Catherine Schaefer, Jordan W. Smoller, Kari Stephansson, Henning Tiemeier, Rudolf Uher, Thomas Werge, Ashley R. Winslow, Gerome Breen, Douglas F. Levinson, Cathryn M. Lewis, Naomi R. Wray, Patrick F. Sullivan, John McGrath, Ian B. Hickie, Narelle K. Hansell, Margaret J. Wright, Nathan A. Gillespie, Andreas J. Forstner, Thomas G Schulze, Stefan Wüst, Markus M. Nöthen, Markus R. Baumgartner, Brian R. Walker, Andrew A. Crawford, Lucía Colodro-Conde, Sarah E. Medland, Nicholas G. Martin, Marcella Rietschel

**Affiliations:** 1 University Hospital of Child and Adolescent Psychiatry and Psychotherapy, Research Department, University of Bern, Bern, Switzerland; 2 SRH University Heidelberg, Academy for Psychotherapy, Heidelberg, Germany; 3 Department of Genetic Epidemiology in Psychiatry, Central Institute of Mental Health, Medical Faculty Mannheim, University of Heidelberg, Mannheim, Germany; 4 Genetics & Computational Biology Department, QIMR Berghofer Medical Research, Brisbane, Australia; 5 Queensland Brain Institute, University of Queensland, Brisbane, Australia; 6 Zurich Institute of Forensic Medicine, Centre for Forensic Hair Analysis, University of Zurich, Zurich, Switzerland; 7 Queensland Centre for Mental Health Research, The Park Centre for Mental Health, Wacol, Australia; 8 National Centre for Register-Based Research, Aarhus University, Aarhus, Denmark; 9 Brain and Mind Centre, University of Sydney, Sydney, Australia; 10 Centre for Advanced Imaging, University of Queensland, Brisbane, Australia; 11 Virginia Institute for Psychiatric and Behavioral Genetics, Virginia Commonwealth University, Richmond, VA, USA; 12 Institute of Human Genetics, University of Bonn, Bonn, Germany; 13 Life & Brain Center, Department of Genomics, University of Bonn, Bonn, Germany; 14 Department of Psychiatry (UPK), University of Basel, Basel, Switzerland; 15 Human Genomics Research Group, Department of Biomedicine, University of Basel, Basel, Switzerland; 16 Department of Psychiatry and Behavioral Sciences, Johns Hopkins University, Baltimore, USA; 17 Institute of Psychiatric Phenomics and Genomics (IPPG), Medical Center of the University of Munich, Campus Innenstadt, Munich, DE Germany; 18 Human Genetics Branch, NIMH Division of Intramural Research Programs, Bethesda, USA; 19 Department of Psychiatry and Psychotherapy, University Medical Center Göttingen, Goettingen, DE Germany; 20 Institute of Experimental Psychology, University of Regensburg, Regensburg, Germany; 21 British Heart Foundation Centre for Cardiovascular Science, Queen’s Medical Research Institute, University of Edinburgh, Edinburgh, UK; 22 Medical Research Council Integrative Epidemiology Unit, School of Social and Community Medicine, University of Bristol, Bristol, UK; 23 MRC Human Genetics Unit, Institute for Genetics and Molecular Medicine, University of Edinburgh, Edinburgh, EH4 2XU UK; 24 Department of Epidemiology, Erasmus Medical Centre, Rotterdam, Netherlands; 25 Psychiatry, Dokuz Eylul University School Of Medicine, Izmir, TR Turkey; 26 Centre for Population Health Sciences, Institute for Genetics and Molecular Medicine, University of Edinburgh, Edinburgh, EH8 9AG UK; 27 Internal Medicine, Erasmus MC, Rotterdam, NL Netherlands; 28 Institute of Behavioural Sciences, University of Helsinki, Helsinki, Finland; 29 National Institute for Health and Welfare, Helsinki, Finland; 30 Institute for Molecular Medicine Finland (FIMM), University of Helsinki, Helsinki, Finland; 31 Department of Medical Genetics, University of Helsinki and University Central Hospital, Helsinki, Finland; 32 Department of General Practice and Primary Health Care, University of Helsinki, Helsinki, Finland; 33 Helsinki University Central Hospital, Unit of General Practice, Helsinki, Finland; 34 Folkhalsan Research Centre, Helsinki, Finland; 35 Vasa Central Hospital, Vasa, Finland; 36 Institute of Health Sciences and Biocenter Oulu, University of Oulu, Oulu, Finland; 37 Department of Children and Yond People and Families, National Institute for Health and elfare, Oulu, Finland; 38 Department of Epidemiology and Biostatistics, MRC-HPA Centre for Environment and Health, Imperial College London, London, UK; 39 Unit of Primary Care, Oulu University Hospital, Oulu, Finland; 40 MRC Centre for Causal Analyses in Translational Epidemiology, School of Social and Community Medicine, University of Bristol, Bristol, UK; 41 School of Social and Community Medicine, University of Bristol, Bristol, UK; 42 Longitudinal Studies Section, Clinical Research Branch, National Institute on Aging, Baltimore, MD, USA; 43 Department of Psychiatry, VU University Medical Center/GGZ inGeest, Amsterdam, Netherlands; 44 Geriatric Unit, ASF, Florence, Italy; 45 University of Groningen, University Medical Center Groningen, Department of Cardiology, Groningen, Netherlands; 46 University of Groningen, University Medical Center Groningen, Department of Genetics, Groningen, Netherlands; 47 Durrer Center for Cardiogenetic Research, ICIN-Netherlands Heart Institute, Utrecht, Netherlands; 48 University of Groningen, University Medical Center Groningen, Interdisciplinary Center for Psychiatric Epidemiology, Groningen, Netherlands; 49 University of Groningen, University Medical Center Groningen, Department of Internal Medicine, Groningen, Netherlands; 50 Wellcome Trust Centre for Human Genetics, University of Oxford, Oxford, UK; 51 Department of Medical Sciences, Uppsala University, Uppsala, Sweden; 52 Samuel Lunenfeld Research Institute, Mount Sinai Hospital, Toronto, Ontario, Canada; 53 School of Women’s and Infant’s Health, The University of Western Australia, Crawley, Australia; 54 Department of Physiology, University of Toronto, Toronto, Ontario, Canada; 55 Center for Bone and Arthritis Research, Institute of Medicin, Sahlgrenska Academy, University of Gothenburg, Gothenburg, Sweden; 56 Child and Adolescent Psychiatry, Erasmus MC, Rotterdam, Netherlands; 57 Psychiatry, Erasmus MC, Rotterdam, Netherlands; 58 Medical and Population Genetics, Broad Institute, Cambridge, USA; 59 Analytic and Translational Genetics Unit, Massachusetts General Hospital, Boston, USA; 60 Department of Psychiatry and Psychotherapy, Universitätsmedizin Berlin Campus Charité Mitte, Berlin, Germany; 61 Department of Biomedicine, Aarhus University, Aarhus, Denmark; 62 iSEQ, Centre for Integrative Sequencing, Aarhus University, Aarhus, Denmark; 63 iSPYCH, The Lundbeck Foundation Initiative for Integrative Psychiatric Research, Aarhus, Denmark; 64 Dept of Biological Psychology, VU University Amsterdam, Amsterdam, Netherlands; 65 Division of Psychiatry, University of Edinburgh, Edinburgh, UK; 66 Centre for Integrated Register-based Research, Aarhus University, Aarhus, Denmark; 67 iPSYCH, The Lundbeck Foundation Initiative for Integrative Psychiatric Research, Aarhus, Denmark; 68 Discipline of Psychiatry, University of Adelaide, Adelaide, Australia; 69 Department of Translational Research in Psychiatry, Max Planck Institute of Psychiatry, Munich, Germany; 70 Munich Cluster for Systems Neurology (SyNergy), Munich, Germany; 71 Department of Psychiatry, Virginia Commonwealth University, Richmond, USA; 72 Center for Neonatal Screening, Department for Congenital Disorders, Statens Serum Institut, Copenhagen, Denmark; 73 Rush Alzheimer’s Disease Center, Rush University Medical Center, Chicago, USA; 74 Institute of Epidemiology and Social Medicine, University of Muenster, Muenster, UK; 75 Human Genetics, Wellcome Trust Sanger Institute, Cambridge, UK; 76 Department of Psychiatry, University Hospital of Lausanne, Prilly, Switzerland; 77 MRC Social Genetic and Developmental Psychiatry Centre, King’s College London, London, UK; 78 University of Oxford, Oxford, UK; 79 Psychological Medicine, Cardiff University, Cardiff, UK; 80 Department of Psychiatry, University of Marburg, Marburg, Germany; 81 Department of Psychiatry, University of Münster, Münster, Germany; 82 Avera Institute for Human Genetics, Sioux Falls, USA; 83 Centre for Cognitive Ageing and Cognitive Epidemiology, University of Edinburgh, Edinburgh, UK; 84 EMGO+ Institute, VU University Medical Center, Amsterdam, Netherlands; 85 Neurology, Brigham and Women’s Hospital, Boston, USA; 86 Stanley Center for Psychiatric Research, Broad Institute, Cambridge, USA; 87 Department of Psychiatry, Massachusetts General Hospital, Boston, USA; 88 Psychiatric and Neurodevelopmental Genetics Unit (PNGU), Massachusetts General Hospital, Boston, USA; 89 Neuroscience and Mental Health Research Institute, Cardiff University, Cardiff, UK; 90 Division of Endocrinology, Children’s Hospital Boston, Boston, USA; 91 Department of Genetics, Harvard Medical School, Boston, USA; 92 Estonian Genome Center, University of Tartu, Tartu, Estonia; 93 Department of Epidemiology, Harvard T.H. Chan School of Public Health, Boston, USA; 94 Department of Mathematics, Massachusetts Institute of Technology, Cambridge, USA; 95 Department of Psychiatry, Trinity College Dublin, Dublin, Ireland; 96 Genetics and Computational Biology, QIMR Berghofer Medical Research Institute, Brisbane, Australia; 97 Bioinformatics Research Centre (BiRC), Aarhus University, Aarhus, Denmark; 98 Institute of Genetic Medicine, Newcastle University, Newcastle upon Tyne, UK; 99 Danish Headache Centre, Department of Neurology, Rigshospitalet Glostrup, Glostrup, Denmark; 100 Institute of Biological Psychiatry, Mental Health Center Sct. Hans, Mental Health Services Capital Region of Denmark, Roskilde, Denmark; 101 iPSYCH, The Lundbeck Foundation Initiative for Psychiatric Research, Copenhagen, Denmark; 102 Department of Psychiatry, Washington University in Saint Louis School of Medicine, Saint Louis, USA; 103 Interfaculty Institute for Genetics and Functional Genomics, Department of Functional Genomics, University Medicine and Ernst Moritz Arndt University Greifswald, Greifswald, Germany; 104 Roche Pharmaceutical Research and Early Development, Pharmaceutical Sciences, Roche Innovation Center Basel, F. Hoffmann-La Roche Ltd, Basel, Switzerland; 105 Department of Medicine, Harvard Medical School, Boston, USA; 106 Max Planck Institute of Psychiatry, Munich, Germany; 107 Division of Research, Kaiser Permanente Northern California, Oakland, USA; 108 Centre for Addiction and Mental Health, Toronto, Canada; 109 Department of Psychiatry, University of Toronto, Toronto, Canada; 110 Psychiatry & The Behavioral Sciences, University of Southern California, Los Angeles, USA; 111 Department of Endocrinology at Herlev University Hospital, University of Copenhagen, Copenhagen, Denmark; 112 Swiss Institute of Bioinformatics, Lausanne, Switzerland; 113 Institute of Social and Preventive Medicine (IUMSP), Lausanne University Hospital, Lausanne, Switzerland; 114 Division of Psychiatry, University College London, London, UK; 115 Mental Health NHS 24, Glasgow, UK; 116 Division of Psychiatry, Centre for Clinical Brain Sciences, University of Edinburgh, Edinburgh, UK; 117 Statistics, University of Oxford, Oxford, UK; 118 EMGO+ Institute for Health and Care Research, Amsterdam, Netherlands; 119 School of Psychology and Counseling, Queensland University of Technology, Brisbane, Australia; 120 Institute of Molecular and Cell Biology, University of Tartu, Tartu, Estonia; 121 Estonian Biocentre, Tartu, Estonia; 122 Institute for Molecular Biology, University of Queensland, Brisbane, Australia; 123 Medical Genetics, University of British Columbia, Vancouver, Canada; 124 Statistics, University of British Columbia, Vancouver, Canada; 125 DZHK (German Centre for Cardiovascular Research), Partner Site Greifswald, University Medicine, Matthias Nauck, Greifswald, Germany; 126 Institute of Clinical Chemistry and Laboratory Medicine, University Medicine Greifswald, Greifswald, Germany; 127 Mental Health Centre Copenhagen, Copenhagen Universtity Hospital, Copenhagen, Denmark; 128 Institute of Health and Biomedical Innovation, Queensland University of Technology, Brisbane, Australia; 129 MRC Centre for Neuropsychiatric Genetics and Genomics, Cardiff University, Cardiff, UK; 130 Humus, Reykjavik, Iceland; 131 Human Genetics and Computational Biomedicine, Pfizer Global Research and Development, Groton, USA; 132 Department of Medical Epidemiology and Biostatistics, Karolinska Institutet, Stockholm, Sweden; 133 Charles E. Schmidt College of Medicine, Florida Atlantic University, Boca Raton, USA; 134 Medical Epidemiology and Biostatistics, Karolinska Institutet, Stockholm, Sweden; 135 Medical Genetics Section, CGEM, IGMM, University of Edinburgh, Edinburgh, UK; 136 Complex Trait Genetics, VU University Amsterdam, Amsterdam, Netherlands; 137 Clinical Genetics, VU University Medical Center, Amsterdam, Netherlands; 138 Psychiatry, University of Iowa, Iowa City, USA; 139 Solid GT, Boston, USA; 140 Department of Biochemistry and Molecular Biology II, Institute of Neurosciences, Center for Biomedical Research, University of Granada, Granada, Spain; 141 Psychiatry, Icahn School of Medicine at Mount Sinai, New York, USA; 142 Department of Psychiatry, University of Groningen, University Medical Center Groningen, Groningen, Netherlands; 143 Division of Cancer Epidemiology and Genetics, National Cancer Institute, Bethesda, USA; 144 Faculty of Medicine, Department of Psychiatry, School of Health Sciences, University of Iceland, Reykjavik, Iceland; 145 School of Medicine and Dentistry, James Cook University, Townsville, Australia; 146 Institute of Health and Wellbeing, University of Glasgow, Glasgow, UK; 147 deCODE Genetics/Amgen, Reykjavik, Iceland; 148 College of Biomedical and Life Sciences, Cardiff University, Cardiff, UK; 149 Institute for Community Medicine, University Medicine Greifswald, Greifswald, Germany; 150 iPSYCH, The Lundbeck Foundation Initiative for Integrative Psychiatric Research, Copenhagen, Denmark; 151 KG Jebsen Centre for Psychosis Research, Norway Division of Mental Health and Addiction, Oslo University Hospital, Oslo, Norway; 152 Department of Psychiatry, University of California, San Diego, San Diego, USA; 153 Institute for Molecular Bioscience, The University of Queensland, Brisbane, Australia; 154 Roche Pharmaceutical Research and Early Development, Neuroscience, Ophthalmology and Rare Diseases Discovery & Translational Medicine Area, Roche Innovation Center Basel, F. Hoffmann-La Roche Ltd, Basel, Switzerland; 155 Department of Psychiatry and Psychotherapy, University Medicine Greifswald, Greifswald, Germany; 156 Department of Psychiatry, Leiden University Medical Center, Leiden, Netherlands; 157 Virginia Institute of Psychiatric & Behavioral Genetics, Virginia Commonwealth University, Richmond, USA; 158 Psychiatry, Columbia University College of Physicians and Surgeons, New York, USA; 159 Division of Epidemiology, New York State Psychiatric Institute, New York, USA; 160 Computational Sciences Center of Emphasis, Pfizer Global Research and Development, Cambridge, USA; 161 Department of Clinical Medicine, Translational Neuropsychiatry Unit, Aarhus University, Aarhus, Denmark; 162 Institute of Neuroscience and Medicine (INM-1), Research Center Juelich, Juelich, Germany; 163 Department of Biomedicine, University of Basel, Basel, CH Switzerland; 164 Division of Medical Genetics, University of Basel, Basel, CH Switzerland; 165 Centre for Integrative Biology, Università degli Studi di Trento, Trento, Italy; 166 Psychiatry, University of California Los Angeles, Los Angeles, USA; 167 Psychiatry, Kaiser Permanente Northern California, San Francisco, USA; 168 Neuroscience Therapeutic Area, Janssen Research and Development, LLC, Titusville, USA; 169 Psychosis Research Unit, Aarhus University Hospital, Risskov, Aarhus, Denmark; 170 Institute of Translational Medicine, University of Liverpool, Liverpool, UK; 171 Psychiatry, Harvard Medical School, Boston, USA; 172 Psychiatry, Dalhousie University, Halifax, Canada; 173 Institute of Clinical Medicine, University of Copenhagen, Copenhagen, Denmark; 174 Human Genetics and Computational Biomedicine, Pfizer Global Research and Development, Cambridge, USA; 175 Orphan Disease Center, Perelman School of Medicine, University of Pennsylvania, Philadelphia, USA; 176 NIHR BRC for Mental Health, King’s College London, London, UK; 177 Psychiatry & Behavioral Sciences, Stanford University, Stanford, USA; 178 Department of Medical & Molecular Genetics, King’s College London, London, UK; 179 Department of Genetics, University of North Carolina at Chapel Hill, Chapel Hill, USA; 180 Department of Psychiatry, University of North Carolina at Chapel Hill, Chapel Hill, USA

## Abstract

Hair cortisol concentration (HCC) is a promising measure of long-term hypothalamus-pituitary-adrenal (HPA) axis activity. Previous research has suggested an association between HCC and psychological variables, and initial studies of inter-individual variance in HCC have implicated genetic factors. However, whether HCC and psychological variables share genetic risk factors remains unclear. The aims of the present twin study were to: (i) assess the heritability of HCC; (ii) estimate the phenotypic and genetic correlation between HPA axis activity and the psychological variables perceived stress, depressive symptoms, and neuroticism; using formal genetic twin models and molecular genetic methods, i.e. polygenic risk scores (PRS). HCC was measured in 671 adolescents and young adults. These included 115 monozygotic and 183 dizygotic twin-pairs. For 432 subjects PRS scores for plasma cortisol, major depression, and neuroticism were calculated using data from large genome wide association studies. The twin model revealed a heritability for HCC of 72%. No significant phenotypic or genetic correlation was found between HCC and the three psychological variables of interest. PRS did not explain variance in HCC. The present data suggest that HCC is highly heritable. However, the data do not support a strong biological link between HCC and any of the investigated psychological variables.

## Introduction

Research has generated robust evidence that chronic stress is a risk factor for mental disorders^1,2^. Understanding the mechanisms through which stress impacts mental health is therefore an important aim of epidemiological research. A core element of the biological stress response is the hypothalamus-pituitary-adrenal (HPA) axis. HPA axis activity is typically measured according to its end product, the steroid hormone cortisol. While prompt HPA axis activation in response to acute stressors is adaptive, long-term dysregulation of basal HPA axis activity and reactivity can have deleterious effects on physiology^2,3^. Alterations in HPA axis regulation are observed in subjects suffering from psychiatric disorders, and have been suggested as not only a consequence but also as a premorbid vulnerability factor^4,5^. Research has shown that variation in HPA axis regulation is influenced by both environmental and genetic factors^6^. Furthermore, authors have suggested that the effects of genetic and environmental risk factors for psychiatric disorders might be mediated in part via dysregulation of HPA axis activity^2,7^. Investigating if HPA axis regulation shares underlying genetic factors with psychological or psychiatric phenotypes can inform about a true biological link between these, potentially indicating a causal involvement of the HPA axis in the vulnerability for psychiatric disorders.

Cortisol is usually measured in saliva, urine, or blood^8^. However, single cortisol measures are strongly influenced by factors such as circadian rhythm, physical activity, and nutrition. Thus the assessment of long-term alterations in HPA axis regulation requires the meticulous assessment of cortisol at multiple time points. In recent years, the assessment of hair cortisol concentration (HCC) has been established as a marker of long-term cumulative HPA axis activity^9,10^. HCC is usually measured in a 3 centimeter (cm) hair segment cut as close as possible to the scalp. Since hair grows, on average, one cm per month, HCC in a 3 cm sample is considered to reflect cortisol secretion during the preceding 3 month period^11^. Hair cortisol is assumed to reflect free cortisol, which is the biologically active share of cortisol not bound to corticosteroid binding globulin (CBG)^12,13^. Studies investigating the relationship of HCC with cortisol levels in other tissues show the highest correlations (up to r = 0.61) with cumulative or average cortisol measures acquired over several days or weeks^14–17^. While these studies which assessed multiple measurement-points have been mainly carried out using saliva and urine, studies which used serum and plasma blood samples, have been mainly based on single assessments, and the observed correlations were lower or non-significant (e.g. refs^18–20^). Studies in pregnant women^14,21^ and in subjects with Cushing’s syndrome^20,22^ -conditions characterized by pronounced alterations in circulating cortisol- have revealed altered HCC levels, and therefore indicate that HCC is a marker of long-term changes in circulating cortisol. HCC therefore represents an efficient method for the retrospective assessment of long-term cortisol secretion, and thus long-term HPA axis activity.

While HCC would thus appear to be an ideal biomarker for stress-related phenotypes, to date, studies of the association between HCC and measures of stress and mental health have generated inconsistent results (for review see refs^9,10,23^). A recent meta-analysis based on aggregated data from a total of 124 samples, and comprising 10,289 subjects, showed no consistent associations with mood disorders or self-reported perceived stress and depressiveness, but found that stress-exposed groups as a whole exhibit 22% increased HCC^24^. While stressful environmental factors play a major role in HPA axis activation, twin studies have indicated that genetic factors have a substantial impact on the secretion of cortisol, especially morning cortisol. Predominantly moderate heritability estimates have been reported in adults (as reviewed in refs^25–27^) and adolescents^25,27,28^. The observed heritability of HPA axis regulation suggests a contribution of genetically determined biological mechanisms. To date, the only large genome wide association study (GWAS) systematically investigating the genes underlying HPA axis activity used total plasma cortisol levels in the morning: Bolton *et al*. (2014) identified associated genetic variants in a region which contains the genes encoding CBG and α1-antitrypsin^29^. If a strong heritability for HCC could been confirmed, HCC would represent a promising target for genetic studies into long-term HPA axis activity and its relationship to mental health.

The first study on heritability of HCC was conducted in a colony of female vervet monkeys, and heritability estimates of ~30% in high and low stress environments were reported^30^. In humans, Tucker-Drob *et al*. (2017) demonstrated heritability of HCC for the first time by investigating an ethnically and socioeconomically diverse sample of 1070 children and adolescents including 533 twin pairs^31^. The authors showed that 65% of the total variability of HCC was explained by additive genetic effects, and that genetic influences on HCC decreased with age. In subjects with low socioeconomic status, a non-significant trend was observed towards increased genetic influences and reduced shared environmental influences on HCC. The authors hypothesized that genetic influences may be stronger under high stress conditions.

As mentioned above, a genetic overlap between HPA axis activity and psychological or psychiatric phenotypes would suggest an involvement of the HPA axis in the vulnerability to psychiatric disorder. However, previous studies investigating a possible genetic overlap between cortisol secretion and psychological variables have been limited in both number and size: A study in 29 monozygotic female twin pairs suggested around 40–45% of the total variance in morning and evening saliva cortisol levels is shared by monozygotic twins^32^. Furthermore, increased (p = 0.06) mean cortisol levels were observed in those twins with a history of major depression (MDD). Notably, intermediate cortisol levels were observed in twins without history of MDD from pairs discordant for history of MDD, however these observations were not significant. A study conducted in 125 female twin pairs demonstrated a heritability of 55% for neuroticism and a heritability of up to 69% for morning cortisol secretion in saliva^33^. However, the authors found no phenotypic or genotypic association between cortisol levels and neuroticism.

In a previous twin study, the present authors demonstrated that continuous measures of perceived stress, depressive symptoms, and neuroticism were heritable and showed strong phenotypic and genetic correlations in healthy adolescent and young adult twins^34^. In line with this, recent large genome-wide studies have demonstrated that genetic variants make a substantial contribution to the development of neuroticism^35,36^, depressive symptoms^36^, and MDD^37^. These studies have also revealed that these risk variants show partial overlap. In a subsequent pilot study, the present authors measured HCC and the three psychological variables in a sample of 109 children and young adults, which included eight monozygotic- and 21 dizygotic twin pairs^38^. Due to the small sample size, no reliable heritability estimates for HCC could be generated. However, the findings suggested that HCC and the assessed psychological variables may share a common genetic basis.

The present larger study aimed (i) to estimate the heritability of HCC and (ii) to investigate the question of whether HPA axis activity shows a genetic overlap with the continuous psychological measures perceived stress, depressive symptoms, and neuroticism, using formal genetic (twin models), and molecular genetic methods i.e. polygenic risk score (PRS) analyses. PRS provide a quantitative measure of genetic risk or vulnerability for a given trait. PRS estimation uses GWAS results to predict genetic risk for each individual in an independent genotyped sample. Investigations can then be performed to determine whether this risk is associated with potentially related phenotypes. However, while significant associations can be found using this approach, the explained variance is limited. For the purposes of the present study, PRS were calculated based on results from GWAS of HPA axis activity^29^; MDD^37^; and neuroticism^35^. Since the GWAS of HPA axis activity was based on plasma cortisol levels, the association between HCC and this PRS was explored i.e. the association between HCC and the genetic variants influencing concentration of cortisol in another tissue.

## Materials and Methods

### Subjects

Our samples consisted of adolescent and young adult twins from the Brisbane area recruited mainly for studies of the genetics of melanoma risk factors and cognition; we neither selected nor excluded participants for any particular phenotype, nor did we systematically obtain data on medical history and treatment. Hair samples were collected from 674 adolescents/young adults. After exclusion of four hair samples (see *‘hair sampling and HCC analysis’*), the final cohort comprised 671 subjects (age mean = 14.5±2.4 years; range = 10.1–31.1 years; 419 females). The cohort included 116 monozygotic (MZ) and 187 dizygotic (DZ) twin pairs (3 families had two sets of DZ twins), and 14 sets of trizygotic triplets. For the purposes of the present analyses, each set of triplets was considered to be a DZ twin pair with one additional singleton. The cohort included a total of 65 singletons, who were derived from the divided triplets (n = 14) and the siblings of the MZ and DZ twin pairs (n = 51) (for details see Supp. Table 1).

All subjects had participated in at least one phase of the Brisbane Longitudinal Twin Study^39,40^. This ongoing, longitudinal study of adolescent/young adult twins and siblings from the general population of the Brisbane area (Australia), is conducted in several phases and investigates somatic- and mental health and related phenotypes (for details see Supp. Text and Supp. Table 2). For 146 subjects (including 29 MZ and 42 DZ twin pairs), hair samples were collected at two time-points (73 subjects at 12 and 14 years; 73 subjects at 14 and 16 years; for details see Supp. Table 1b). Stability of HCC and the psychological variables was assessed in terms of correlations between the two time points (for details see Supp. Table 1b). For 432 subjects (age mean =  15.5± 2.4 years; range =  10–31 years; 268 females), genome-wide genotype data were available (for details see Supp. Table 1c). The study was approved by the Human Research Ethics Committee of the Queensland Institute of Medical Research (QIMR) and conducted in accordance with the Declaration of Helsinki. Written informed consent was obtained from all subjects, and from legal guardians in the case of minors, prior to inclusion and sample collection.

### Psychological variables

For participants less than 16 years old, perceived stress was measured using the 30-item ‘Daily Life and Stressors Scale’ (DLSS^41^) and neuroticism was measured using the respective 20 items of the ‘Junior Eysenck Personality Questionnaire’ (JEPQ^42^) as the DLSS and JEPQ are validated for children and adolescents. For participants aged 16 years or older, perceived stress was measured using the 10-item ‘Perceived Stress Scale’ (PSS^44^). Additionally, 168 subjects between 16 and 19 years completed both the PSS and the DLSS, and this overlap was used to harmonize the two scales using item response theory (IRT; see below). For participants aged 16 years or older, neuroticism was measured using the respective 12 items of the ‘NEO-Five Factor Inventory revised version’ (NEO-FFI-R^44^). At all ages, depressive symptoms were assessed using the 34-item ‘Somatic and Psychological Health Report’ (SPHERE;^45^). For all subjects, measures of stress (PSS or DLSS), neuroticism, and depressive symptoms were obtained at the time of hair sampling. For some subjects, data were unavailable for perceived stress (n = 1); neuroticism (n = 51); and depressive symptoms (n = 55), since subjects did not fill out the respective questionnaires. In the subgroup that underwent assessment at two time-points, one subject had missing data for perceived stress and depressive symptoms at the second time point.

### Hair sampling and HCC analysis

Using fine scissors, a 3 cm hair swatch of approximately 3 millimeters in diameter was cut as close as possible to the skin from the posterior vertex of the scalp. Hair cortisol was analyzed by TMB and MRB at the Institute of Forensic Medicine, Centre for Forensic Hair Analysis, University of Zurich. Cortisol concentration was measured using liquid chromatography-tandem mass spectrometry (LC-MS/MS), as described by Binz *et al*.^46^ (for details see Supp. Text). Prior to HCC measurement, hair samples were randomly assigned to batches (irrespective of time-point or twin-pair) in order to minimize the effect of batch differences on twin correlations. To assess technical error, 106 hair samples were assessed in duplicate and 27 in triplicate. The hair samples were assayed between April and July 2016 in a total of 35 batches (comprising 27 samples respectively). Samples with extreme high or low values (n = 26; 13 ≤ 0.2 and 13 ≥ 26.3) were re-assayed in order to confirm the extreme values.

### Statistical Analysis

#### Treatment of psychological variables and HCC

To harmonize neuroticism scores, the neuroticism sum-scores of the NEO-FFI-R and the JEPQ scores were separately z-transformed and then combined, as described in previous studies^47,48^. To harmonize data from the two stress rating scales DLSS and PSS, Item Response Theory (IRT) analysis was performed (for raw values and details see Supp. Text and Supp. Table 3). IRT models have the advantage that the difficulty and discriminability of each item is taken into account by modeling a normally distributed liability based on the responses to the individual questionnaire items. It is thus superior to a simple sum score that assumes all items have the same discriminating ability with respect to the underlying liability being measured and is thus particularly useful if widely different scales are being combined–as here for perceived stress. We made use of the overlapping cases who had completed both scales in order to put both measures on the same liability scale. IRT analysis was also applied to the 34 items from the SPHERE in order to produce a single liability measure for depressive symptoms.

To account for skewness, raw HCC values (mean = 6.29 pg/mg; SD = 28.92; range = 0–560) were log transformed. Here, the lowest measured value (0.1) was added to each value prior to log_10_ transformation. Five outliers>3SD (HCC>64.70 pg/mg) were winsorized to 3SD on the lg_10_ scale. Analyses were also conducted to test the effect of experimental variables reported in previous studies of HCC. The analyses included: (i) batch number (n = 35); (ii) study phase (n = 6); (iii) storage time, defined as time between date of collection and date of assay (range = 0.50–4.02 years) and divided into n = 5 groups according to increasing storage time; and (iv) sun exposure, operationalized according to month of assessment (n = 12) and self- and maternal ratings of sun exposure. Since all aforementioned variables changed the fit of the model significantly, they were regressed out from the HCC measurement using a linear model, including the dummy coded variables as fixed effects. Subsequent analyses were carried out with the residuals of this model. The HCC value from the first time point was used in the following analyses; the second time point was only used for assessing stability of HCC over a two-year period in the subset of longitudinally assessed subjects (n = 146).

#### Twin correlations, heritability of HCC, and shared covariance with psychological variables

To estimate twin correlations and the heritability of HCC and the three psychological variables of interest, the twin and sibling data were used to generate structural equation models. Model parameters were estimated using the full maximum likelihood method implemented in Mx^49^. This makes use of all data points - including those of unpaired twins and singletons - in order to improve estimation of sample means and variances. This approach allows partitioning of the variation into: additive genetic influences (A); shared environmental influences (C); and unique environmental influences (E). Using likelihood ratio chi-square tests, sub-models with only two factors (AE and CE models) were compared with the three-factor models (ACE) in order to estimate the sources of variance and select the most parsimonious variance structure of the traits.

To investigate the influence of genetic and environmental factors on HCC and the psychological variables of interest, as well as genetic and environmental correlations between these variables, multivariate analysis was performed. This involved use of a simultaneous Cholesky decomposition. A Cholesky decomposition is a good initial multivariate method to use in the absence of a clear model of the factor structure relating a set of correlated variables. Furthermore, taking advantage of having both MZ and DZ twins one can fit a 3-part Cholesky model including decompositions of (co)variance due to A (additive genetics), C (shared environment) and E (unique environment), since one cannot assume that factor loadings between these three sources of variation will be proportional for each of the examined variables. In fact, there are known cases of opposite-signed loadings of factors A and C (e.g., for items of Eysenck’s Psychoticism scale^50^). The source of variation, C, captures cultural environment shared by twins within a family regardless of zygosity and could include factors such as sharing the same school, neighborhood, and exposure to infectious diseases. C is distinguished from unique environment, E, which is specific to individuals and may include factors such as accidents; however, it is important note that E also includes measurement error, which is often the major contributor to this source of variance. An ACE Cholesky decomposition model was compared with an AE and a CE Cholesky decomposition. Perceived stress was used as the first, depressive symptoms as the second, neuroticism as the third, and HCC as the last latent factor in order to estimate (i) the genetic variance of HCC that is shared with genes affecting the psychological variables and (ii) the independent genetic variance for HCC after removing the effects of the genes with the primary influence on the psychological variables (for further details see Supp. Text).

The fit of each model was assessed according to the differences in log likelihood between the sub and the full models. The most parsimonious model was chosen for the purposes of data interpretation. Sex and puberty which - generally starts earlier in girls- influence the cortisol secretion^51^. As puberty status was not assessed in the study, sex, age, age², sex x age, and sex x age² were included as covariates in all models, to allow for different age effects in boys and girls and the fact that these may be curvilinear. Body mass index (r = 0.06, p = 0.11), socio-economic index (r = -0.07, p = 0.13), and hair dyeing (r = 0.01, p = 0.81) showed no significant associations with HCC in the present sample and were thus not included as covariates. Since previous results suggest that heritability of HCC is age-dependent^31^, a separate analysis was performed in the younger and the older half of the sample, as defined by a median split to test the heritability of HCC in the different age groups. Further details of the twin design and analytical methods, including assumption testing and multivariate modeling, are provided elsewhere^52^.

#### Genotyping, quality control, and imputation

Genotyping was performed using the Illumina Human610-Quad and Core+Exome SNP chips. Quality control included inspection of pedigree, sex, Mendelian errors, and ancestry, as well as filtering for genotyping quality (GenCall <0.7); SNP and individual call rates (<0.95); Hardy-Weinberg equilibrium failure (P <10–6); and minor allele frequency (<0.01). Subjects were imputed to the Haplotype Reference Consortium (HRC.1.1)^53^ on the Michigan Imputation Server (https://imputationserver.sph.umich.edu/i). Imputation was carried out in two separate waves for the Illumina Human610-Quad and the Core+Exome SNP chips. To account for population stratification (i.e. allele frequency differences between subjects due to systematic ancestry differences) in the PRS analysis, genetic principal components (PC) reflecting the respective ancestry were calculated using EigenSoft 6.0.1 (http://www.hsph.harvard.edu/alkes-price/software/). Further details on genotyping, quality control, and imputation are provided in the Supp. Text.

#### Polygenic risk score analysis

PLINK 1.90 (version 3, May 2016, https://www.cog-genomics.org/plink2/) was used to compute PRS in accordance with the procedure described by Wray *et al*.^53^. For PRS estimation, the results of large GWAS (discovery sample) are used to calculate an aggregated genetic risk score for each individual in an independent genotyped sample (target sample). The PRS represents the sum of the risk alleles, as weighted by their respective estimated effect sizes. PRS provide a quantitative measure of the genetic risk or vulnerability for a given trait: The higher the score, the higher the predisposed genetic risk of the individual for the trait in question. In the present study, PRS were calculated using summary statistics from recent GWAS or GWAS meta-analyses of: (i) plasma cortisol (CORtisol NETwork (CORNET) Consortium^29^, comprising 12,597 subjects); (ii) MDD (PGC-MDD2^37^ minus QIMR samples, comprising a total of 49,524 cases and 110,074 controls, for details see Supp. Table 4); and (iii) neuroticism (UK Biobank^35^, comprising 91,370 subjects). The SNP-sets used to compute the PRS were selected using eight different p-value thresholds (5e-8, 1e-5, 0.001, 0.01, 0.05, 0.1, 0.5, 1.0) in the respective discovery sample.

To take family structure into account, associations of PRS with HCC and the three psychological variables of interest were tested using linear mixed regression models in GCTA (Genome-wide Complex Trait Analysis v. 1.26)^55^. Here, the following were used as covariates: sex; age; age²; sex *x* age; sex *x* age²; the first five genetic principal components (PC); and the imputation wave. One-sided p-values are reported, according to the hypothesis of a positive association of the PRS with HCC and each of the psychological variables. Further details of the PRS analysis are provided in the Supp. Text.

#### Data availability

The datasets generated during and/or analyzed during the current study are not publicly available due to privacy regulations but are available from the authors on reasonable request.

## Results

### Clinical characteristics of the cohort

HCC was measured in 671 subjects. HCC, neuroticism and depressive symptom scores were higher in females than in males. Furthermore, perceived stress, depressive symptoms and neuroticism showed an association with age, and perceived stress and neuroticism showed an interaction of sex and age. Details can be found in Supp. Table 5. Distributions for age and all variables are shown in Table 1.

**Table 1:** Means (SD) for age, psychological variables and HCC.

	Total (n = 671)	Male (n = 252)	Female (n = 419)
Age (years)	14.49 (2.43)	14.42 (2.59)	14.53 (2.32)
Perceived stress^a^	0.01 (0.58)	−0.02 (0.61)	0.03 (0.57)
Depressive symptoms^b^	0.01 (0.92)	−0.09 (0.94)	0.07 (0.90)
Neuroticism^c^	0.01 (1.01)	−0.12 (1.02)	0.08 (0.99)
Hair cortisol (pg/mg)	5.31 (14.23)	5.68 (18.42)	5.09 (10.98)

Abbreviations: SD = standard deviation. ^a^IRT-scores deriving from perceived stress questionnaires (DLS and PSS); ^b^IRT-scores of the SPHERE ^c^z-standardized values of the neuroticism questionnaires.

### Influence of experimental covariates on HCC

Quality control of HCC assessed in duplicate and triplicate revealed a high correlation of the log transformed HCC values for samples re-assayed once (n = 106; r = 0.89) and samples assayed three times (n = 27; r = 0.98 with the 1^st^ and r = 0.98 with the 2^nd^ assay).

Analysis of the influence of experimental covariates resulted in a final model with a total of 54 deviations for experimental effects (34 for batch number, 4 for storage time, 11 for month, and 5 for the respective study phase). All of these experimental covariates were highly significant. Notably, the analysis demonstrated: (i) a decrease in HCC with increasing storage time; and (ii) maximum values in March (end of the Australian summer) and minimum values in September (end of the Australian winter). Dropping any one of the covariates from the full model (which includes all four) increased variance by 5.9% for batch effects, 1.8% for storage effects, 3.8% for month effects, and 6,4% for study effects (details see Supp. Table 6).

### Heritability

For HCC, a DZ correlation of r = 0.42, and a MZ correlation of r = 0.66 were observed. This corresponded to a heritability estimate of h² = 0.72 in the multivariate model including HCC and the psychological variables. Slightly lower DZ and MZ twin correlations were observed for the psychological variables. Here, the heritability estimates were h² = 0.54 for perceived stress; h² = 0.55 for depressive symptoms; and h² = 0.56 for neuroticism (see Table 2).

**Table 2:** Monozygotic (MZ) and dizygotic (DZ) twin correlations (95% CI), heritability and stability over two years, for the psychological variables and HCC.

	rMZ (115 pairs)	rDZ (183 pairs)	Heritability^a^	Two year Stability^b^ (n = 146)
Perceived stress	0.51 (0.37–0.61)	0.29 (0.17–0.40)	0.54 (0.42–0.64)	0.61 (0.50–0.69)
Depressive symptoms	0.55 (0.43–0.65)	0.27 (0.13–0.40)	0.55 (0.42–0.65)	0.51 (0.37–0.62)
Neuroticism	0.53 (0.42–0.62)	0.31 (0.17–0.44)	0.56 (0.45–0.67)	0.58 (0.46–0.67)
Hair cortisol	0.66 (0.56–0.74)	0.42 (0.31–0.51)	0.72 (0.63–0.79)	0.32 (0.16–0.45)

Abbreviations: CI = confidence interval, rDZ = correlation between dizygotic twins, rMZ = correlation between monozygotic twins. All calculations were corrected for sex, age, age^2^, sex x age, sex x age^2^. ^a^Heritabilities were estimated using the multivariate model (see Fig. 1). ^b^Two year stability was calculated as the correlation between time point 1 and time point 2.

### Stability over time

As shown in Table 2, stability over the two-year period ranged between r = 0.51 and r = 0.61 for the psychological variables, while HCC stability was r = 0.32.

### Correlation of HCC with psychological variables

High phenotypic correlations were found between the three psychological measures, ranging from r = 0.59 to r = 0.64 (Supp. Table 7). In contrast, negligible correlations were observed between the three psychological measures and HCC, with r = 0.04 for perceived stress; r = 0.07 for depressive symptoms; and r = 0.08 for neuroticism. None of these correlations differed significantly from zero.

**Figure 1:**
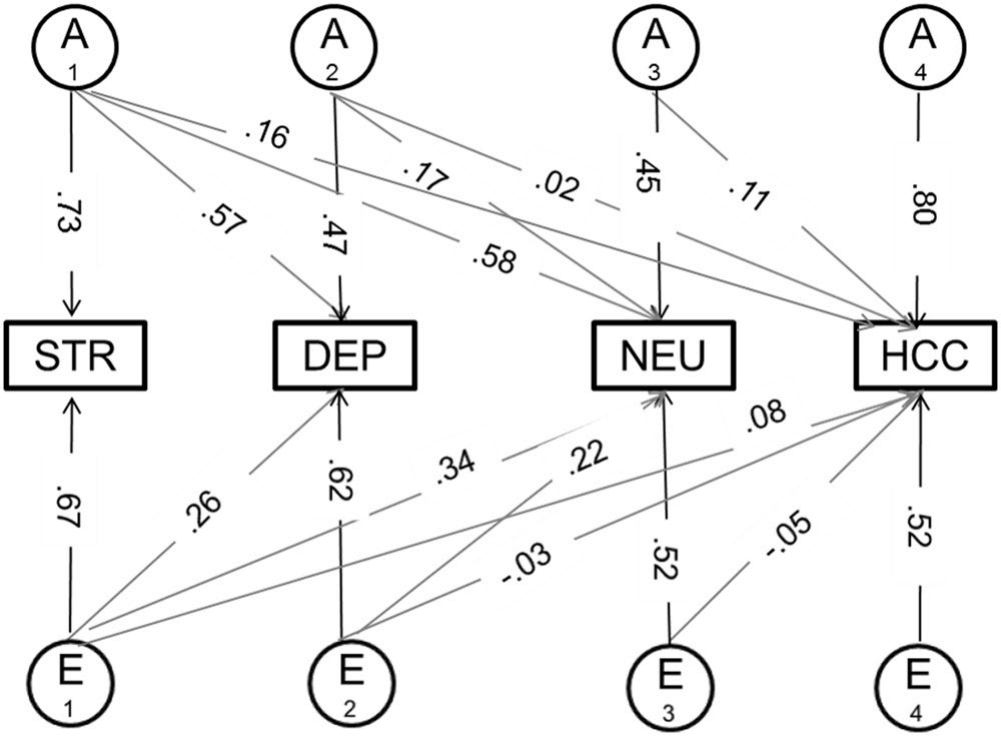
Cholesky Decomposition for the AE Model. Latent factor loadings are standardized to unit variance and must be squared to obtain standardized variance components. A1-A4 additive genetic factors, E1-E4 unique environmental factors. Abbreviations: STR = perceived Stress, DEP = depressive symptoms, NEU = neuroticism, HCC = hair cortisol concentration.

As counterbalancing genetic and environmental correlations of opposite sign resulting in a small or zero phenotypic correlation have been observed for psychological measures (e.g. psychoticism)^49^, these associations were further explored with a multivariate Cholesky decomposition (Fig. 1). The C matrix, which accounts for shared environmental influences, could be dropped from the model without worsening fit (Δc^2^ = 13.66, p = 0.19). The AE model revealed low and non-significant genetic correlations (r_A_) between HCC and the three psychological variables (r_A_ = 0.14 for perceived stress; r_A_ = 0.12 for depressive symptoms; r_A_ = 0.19 for neuroticism) (Table 3).

**Table 3:** Results of the Cholesky Decomposition for the AE Model, with (I) Standardized Parameters and (II) Genetic and environmental correlations between HCC and psychological measures.

		(I) Standardized Parameters %				(II) Correlations			
		1	2	3	4	1	2	3	4
Genetic (A)	1-Perceived stress	53.89				1			
	2-Depressive symptoms	32.95	22.10			0.77	1		
	3-Neuroticism	33.78	2.79	19.81		0.77	0.74	1	
	4-Hair cortisol	1.33	0.03	1.20	69.37	0.14	0.12	0.19	1
Unshared Environment (E)	1-Perceived stress	46.12				1			
	2-Depressive symptoms	6.65	38.30			0.35	1		
	3-Neuroticism	12.15	5.00	26.48		0.53	0.52	1	
	4-Hair cortisol	0.68	0.07	0.21	27.02	0.16	0.11	0.19	1

Separate AE Cholesky analysis was then performed for the younger (mean age = 12.37 (SD = 0.54) (Supp. Table 8), and the older (mean age = 15.70 (SD = 2.32)) (Supp. Table 9) halves of the cohort (divided by median; Mdn = 14.01 years). Comparable heritability estimates for HCC were found in the younger (h^2^ = 0.74) and older (h^2^ = 0.69) halves of the cohort. Heritabilities for the psychological variables were also broadly consistent between the younger and older halves of the sample (see Supp. Tables 8 and 9).

### Association of polygenic risk for plasma cortisol, MDD, and neuroticism with HCC and psychological variables

**Figure 2:**
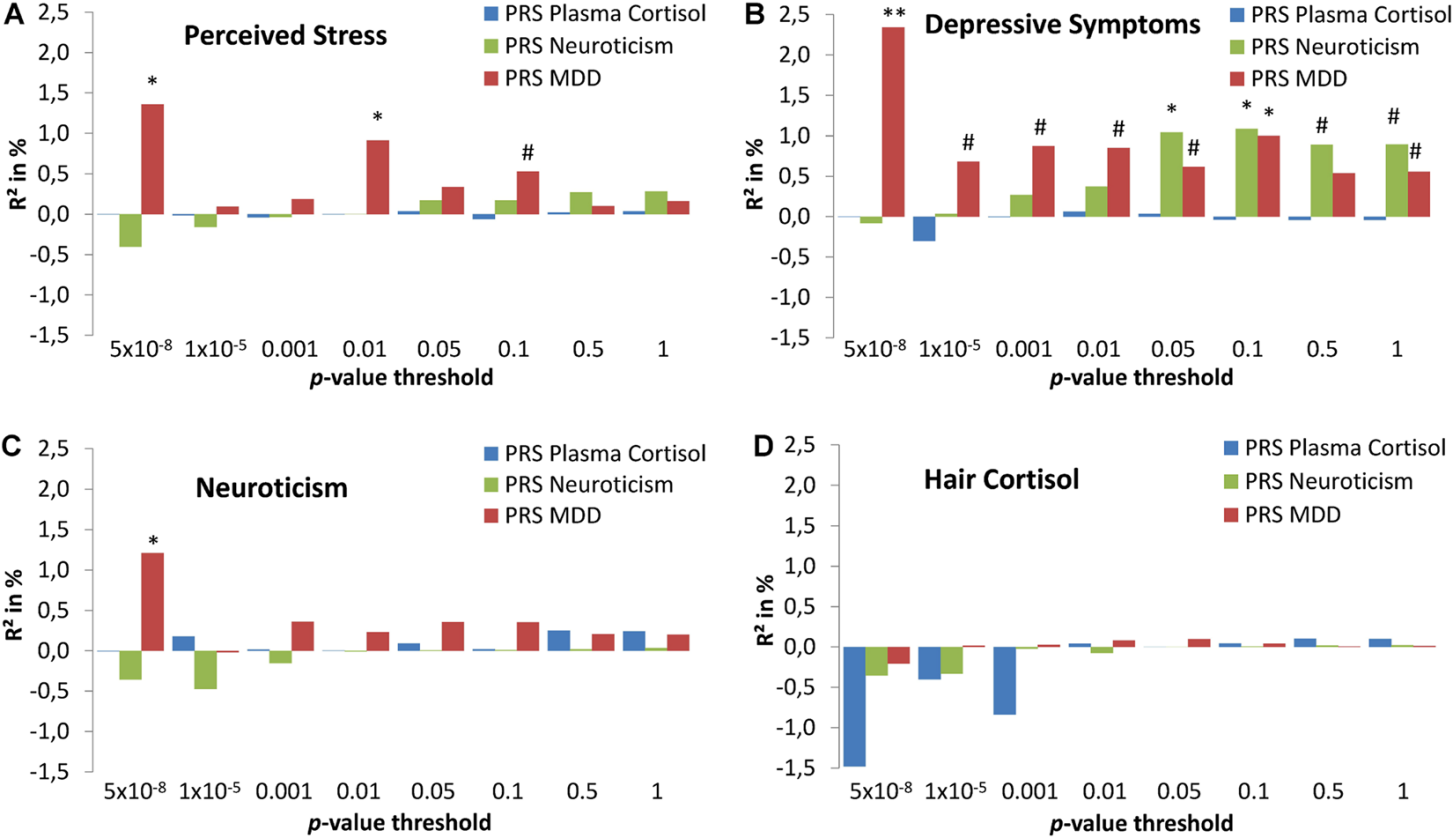
Association of polygenic risk scores (PRS) for plasma cortisol, neuroticism, and major depression (MDD) with: (**A**) perceived stress; (**B**) depressive symptoms; (**C**) neuroticism; and (**D**) hair cortisol concentration. Negative R² indicates a negative direction of the association of PRS with the respective phenotype; p one-sided: **p < 0.01; *p < 0.05; ^#^p < 0.1.

The PRS for plasma cortisol showed no significant association with the HCC at any of the chosen thresholds (Fig. 2D). Furthermore, the PRS for plasma cortisol did not predict any of the three psychological variables (Fig. 2A–C). The PRS for MDD and the PRS for neuroticism showed positive associations with the psychological variables. For several p-value thresholds, in particular thresholds>0.01, these associations reached nominal significance (see Fig. 2A–C). No significant association was found between HCC and the PRS for MDD or the PRS for neuroticism (Fig. 2D). Details of the PRS regression analyses are provided in Supp. Tables 10–21.

## Discussion

To our knowledge, the present study is the first to assess the heritability of HCC together with its phenotypic and genetic association with perceived stress, depressive symptoms, and neuroticism.

The analyses generated a heritability estimate for HCC of ~70%, with no significant contribution being found for shared environment. These estimates are nearly identical to those reported by Tucker-Drob and colleagues^31^ and at the upper end of those reported for measures of cortisol in other tissues (e.g. saliva and urine). This is not surprising, given that HCC is an integrated, rather than a point measure of HPA axis activity. It can now be taken as a fact that in adolescents from the general population, a substantial proportion of HCC variance is attributable to genetic factors. At first glance, this may appear surprising for a parameter that is considered a potential biomarker for stress. However, this finding does not preclude environmentally induced changes. Furthermore, the stability of HCC, as measured in a subgroup over a two-year period, is relatively low (r = 0.32). These stability measures are comparable to those found in students aged 17–21 years assessed at three time points over one year (r = 0.25–0.39)^56^ and to those found in children aged 1, 3, 5, and 8 years, in whom HCC was assessed over periods of two and three years (r = 0.30–0.44)^57^. In samples with a higher mean age (>30 years), in which intervals of assessment ranged between one month and one year, higher stability measures (r = 0.68−0.86) have been reported^17,58^. These findings are in line with the hypothesis of greater changes of HPA axis functioning during childhood and adolescence which are periods marked by dramatic physical, cognitive, social and emotional changes^56^. Unfortunately, no conclusions can be drawn from the present analyses concerning the heritability of the intra-individual change in HCC over time, since the respective subsample was small and lacked sufficient statistical power for this analysis. However, it is of interest to note, that the heritability estimate of HCC did not differ between the younger and older half of the sample.

Regarding the phenotypic and genetic correlations of HCC with psychological variables, the present analyses demonstrated that shared genetic factors underlie the association between the psychological variables perceived stress, depressive symptoms, and neuroticism. The heritability of neuroticism and depression^59–61^, as well as their genetic overlap^62,63^, are well established, and the present data are consistent with those of previous reports.

However, contrary to the results of our previous pilot study^38^, no significant phenotypic or genetic overlap was found between HCC and any of the three psychological variables. Although no study to date has investigated the association between HCC and neuroticism, the present results are consistent with a previous investigation of 125 twin pairs, which showed heritability for neuroticism and morning cortisol secretion in saliva but no phenotypic or genetic overlap between these two variables^33^.

The present findings must be viewed with caution, as they are derived from a cohort of relatively healthy adolescents from the general population. It is possible that the association between HCC and psychological variables may only become apparent for more extreme psychological phenotypes. While studies of subjects with a history of chronic or traumatic psychological disturbance, i.e., conditions which are known to alter HPA axis functioning (e.g., shift work, earthquake, or civil war), have repeatedly reported HCC alterations^64–66^, previous reports on the association between HCC and psychological variables in healthy subjects are inconsistent (e.g. refs^9,67–72^). Recently, this was reflected in a meta-analytic study, which found no evidence for a positive association of HCC with subjective stress and depressivity^24^. However, the meta-analysis confirmed that HCC is increased in conditions of chronic ongoing stress, and therefore represents a marker for the assessment of long-term alterations in cortisol levels in response to environmental influences^24^.

In the present sample, we observed values within the normal range of the applied psychological scales (see Supp. Table 3). If significant alterations in HCC only occur in response to major (and chronic) stressors, genetic and phenotypic correlations between HCC and psychological variables may only become observable in subjects who display more pronounced phenotypes, such as full-blown psychiatric disorders, or who are subjected to extreme levels of stress. Therefore, future genetic studies should investigate groups that are extreme in terms of perceived stress (e.g. after traumatic events), or twin pairs in which at least one twin has severe mental health problems.

Additionally, such studies should aim to further dissect the factors underlying HPA axis deregulation by including -besides basal measures such as HCC- dynamic measures of HPA axis (re-)activity, such as circadian rhythm, and reactivity to psychological, physical and pharmacological challenges, which are commonly assessed using multiple saliva or blood samples within a defined sampling scheme. Those measures have been shown to be altered in psychiatric disorders, partially independent of or even contrary to alterations in basal cortisol levels (e.g. as measured in saliva or blood)^73,74^. Another aspect future studies should address is the interplay of HCC with other hormones known to affect HPA axis activity, which can also be measured in hair, such as cortisone or gonadal steroids.

The molecular genetic approach i.e. using polygenic risk scores to investigate the association between HCC and psychological measures generated several interesting results. First, the observation of nominally significant positive associations of the PRS for MDD and the PRS for neuroticism with the psychological variables is consistent with reported formal genetic correlations. This supports the validity of the approach, even in a sample as small as that used in the present analyses. However, the relatively low degree of explained variance, and the fact that the associations only achieved nominal significance at some of the selected thresholds, highlights that our approach lacks sufficient power. Second, the observation that neither the PRS for MDD nor the PRS for neuroticism predicted HCC, and that the PRS for plasma cortisol did not predict perceived stress, depressive symptoms, or neuroticism, parallels the genetic results from twin models, which suggests a lack of genetic overlap between HCC and the psychological variables. Interestingly, no overlap was found between the PRS for plasma cortisol and HCC. For the interpretation of this result it is important to note, that hair cortisol is assumed to reflect free (unbound) cortisol, while the GWAS investigated total (bound and unbound) plasma cortisol concentrations^12,13^. The main signal of the GWAS was observed in the region coding for CBG, the main regulator of the ratio of free and total cortisol^29^ and might thus differentially affect measures of free and total cortisol. Additionally, HCC represents a measure of accumulated long-term cortisol secretion, while the GWAS was based on a one-time measure of morning plasma cortisol. As described, the correlations between HCC and one time measures of cortisol in saliva and blood are inconsistent. However, in view of the limited power, the results of the PRS analyses must be interpreted with caution, and large future studies of easier-to-recruit, unrelated subjects might generate insights into the associations. Additionally, even though each of the GWAS considered in the present analyses involved cohorts in excess of 10,000 subjects and identified genome-wide genetic variants, larger studies are required. Research has demonstrated that for complex phenotypes, GWAS involving several 100,000 subjects are needed to identify the majority of the common polygenic variation^54,75^. The power of PRS calculated using future GWAS will be increased due to a superior signal to noise ratio in these larger datasets.

## Limitations

The present findings should be interpreted with caution, since the study had several limitations. First, the sample size was relatively small in terms of the establishment of twin models, particularly in the case of the exploratory analyses investigating subsamples divided by age, and the detection of small correlations between HCC measures and psychological phenotypes. Second, generalizability of the results to the general population is limited, since a young and relatively healthy cohort was investigated using self-rating questionnaires. Phenotypic and genetic correlations with psychological variables may only become evident in cohorts with more pronounced or specified environmental impacts (e.g. chronic severe stress) and more extreme phenotypes (e.g. psychiatric disorders, biologically relevant endophenotypes). Under the challenge of more adverse environments, stronger variance might occur in those phenotypes, partially driven by distinct genetic factors. Third, we did control for age and sex and interactions in our analyses. However, there is evidence that the influence of puberty processes on the associations between HPA axis activity with stress and depressive symptoms is best accounted for by assessing pubertal status and timing (e.g. refs^76–78^). Future studies of HCC in adolescents should consider including those measures. Fourth, the self-rating questionnaires for perceived stress and depression do address shorter time frames (last week to last few weeks) than the time frame reflected in the 3 cm segments of hair analyzed for hair cortisol (~3 months). However, we observed a high heritability and stability (over two years) of the psychological measures in our sample. This indicates that these measures largely assess more stable components of the underlying psychological constructs and not merely short term fluctuations. Here, however, we must acknowledge the difficulty for researchers in this area of finding state effects convincingly independent of trait disposition. Fifth, the PRS scores for neuroticism, MDD, and plasma cortisol were derived from adult cohorts, and statistical power was small due to the limited size of the learning and present cohorts. Replication studies in much larger cohorts are required before further conclusions can be drawn, in particular as regards the genetic overlap between plasma cortisol and HCC. Sixth, although the present analyses demonstrate that HCC can be assessed with a high degree of reliability, the quality control analysis demonstrated that various factors influenced HCC, including batch number, season, storage time, and study phase, and had to be statistically controlled for. Fifth, these corrections may have contributed to an overcorrection, and thus to the overlooking of true findings. Seventh, we did not systematically obtain data on medical history or treatment, including oral contraceptives. However, as the major part of our sample is less than 16 years of age, it is unlikely that oral contraceptives and other medications represent a major confounder. Furthermore, unlike cortisol assessed in other tissues^6^, there is no strong evidence that oral contraceptives have a substantial effect on HCC (e.g. refs^23,24,79^). Eighth, hair dyeing did not affect HCC in the present sample. However, further variables such as frequency of hair washing were not assessed.

## Conclusions

Our study demonstrates a high heritability of HCC, but no evidence for a genetic overlap with depressive symptoms, perceived stress and neuroticism. HCC is a reliable measure of long-term HPA axis activity and genetic effects play a major role in inter-individual variability. This knowledge will inform future research, and is of particular relevance in terms of the interpretation of data from cross-sectional studies. If a genetic or phenotypic correlation does exist between HCC and perceived stress, depressive symptoms, or neuroticism, the present analyses have demonstrated that this is difficult to identify in a relatively small sample of young adults from the general population. Further studies are warranted to investigate whether this is also the case in samples with more extreme psychological phenotypes. Particularly in studies for which blood or saliva sampling over several days and multiple time-points is difficult to implement, HCC represents a promising alternative measure for the assessment of long-term HPA axis activation. The high heritability, easy accessibility and cost-effectiveness of HCC render it a promising target for future large scale GWAS of the biological pathways that underlie long-term cortisol secretion and its links to stress-related phenotypes.

## Electronic supplementary material

Supplementary Material
