# Supplementary material for: Hair Cortisol in Twins: Heritability and Genetic Overlap with Psychological Variables and Stress-System Genes
Source: Sci Rep. 2017 Nov 10;7:15351. doi: 10.1038/s41598-017-11852-3 (PMC5703444; doi:10.1038/s41598-017-11852-3)
Supplement: Supplementary file 1 — Supplementary Material [file 41598_2017_11852_MOESM1_ESM.pdf]

Supplementary Material

Hair Cortisol in Twins: Heritability and Genetic Overlap with Psychological Variables and Stress-System Genes

Liz Rietschel[1*], PhD; Fabian Streit[2$*], Dipl.-Psych.; Gu Zhu[3], PhD; Kerrie McAloney[3]; Josef Frank[2$], Dipl.-Psych.; Baptiste Couvy-Duchesne[3,4$], MSc; Stephanie H. Witt[2$], PhD; Tina Binz[5], PhD; CORtisolNETwork (CORNET) Consortium[#]; Major Depressive Disorder Working Group of the Psychiatric GWAS Consortium (PGC)[#]; John McGrath[4,6,7$], MD; Ian B. Hickie, PhD[8$]; Narelle K. Hansell[4], PhD; Margaret J. Wright[4,9], PhD; Nathan Gillespie[3,10], PhD; Andreas J. Forstner[11,12,13,14$], MD; Thomas G Schulze[2,15,16,17,18$], MD; Stefan Wüst[2,19], PhD; Markus M. Nöthen[11,12$], MD; Markus Baumgartner[5], PhD; Brian R. Walker[20§], MD; Andrew A. Crawford[20,21§], PhD; Lucia Colodro Conde[3$], PhD; Sarah E. Medland[3$], PhD, Nicholas G. Martin[3$**], PhD; Marcella Rietschel[2$**], MD

[*]shared first authorship

[**]shared senior authorship

[$]member of Major Depressive Disorder Working Group of the PGC

[§]member of CORNET

[#]further consortium members are listed at the end of the main manuscript

Institutional affiliations:

[1]University Hospital of Child and Adolescent Psychiatry and Psychotherapy, Research Department, University of Bern, Bern, Switzerland

[2]Department of Genetic Epidemiology in Psychiatry, Central Institute of Mental Health, Medical Faculty Mannheim, University of Heidelberg, Mannheim, Germany

[3]Genetics & Computational Biology Department, QIMR Berghofer Medical Research, Brisbane, Australia

[4]Queensland Brain Institute, University of Queensland, Brisbane, Australia

[5]Institute of Forensic Medicine, Centre for Forensic Hair Analysis, University of Zurich, Zurich, Switzerland

[6]Queensland Centre for Mental Health Research, The Park Centre for Mental Health, Wacol, Australia

[7]National Centre for Register-Based Research, Aarhus University, Aarhus, Denmark

[8]Brain and Mind Centre, University of Sydney, Sydney, AU

[9]Centre for Advanced Imaging, University of Queensland, Brisbane, Australia

[10]Virginia Institute for Psychiatric and Behavioral Genetics, Virginia Commonwealth University, Richmond, VA, USA.

[11]Institute of Human Genetics, University of Bonn, Bonn, Germany

[12]Life&Brain Center, Department of Genomics, University of Bonn, Bonn, Germany

[13]Department of Psychiatry (UPK), University of Basel, Basel, Switzerland

[14]Human Genomics Research Group, Department of Biomedicine, University of Basel, Basel, Switzerland

[15]Department of Psychiatry and Behavioral Sciences, Johns Hopkins University, Baltimore, US

[16]Institute of Psychiatric Phenomics and Genomics (IPPG), Medical Center of the University of Munich, Campus Innenstadt, Munich, DE

[17]Human Genetics Branch, NIMH Division of Intramural Research Programs, Bethesda, US

[18]Department of Psychiatry and Psychotherapy, University Medical Center Göttingen, Goettingen, DE

[19]Institute of Experimental Psychology, University of Regensburg, Germany

[20]British Heart Foundation Centre for Cardiovascular Science, Queen's Medical Research Institute, University of Edinburgh, Edinburgh, UK

[21]Medical Research Council Integrative Epidemiology Unit, School of Social and Community Medicine, University of Bristol, Bristol, United Kingdom

Supplementary Text:

Subjects

All subjects had participated in various phases of the Brisbane Longitudinal Twin Study. These phases include the Brisbane Twin Nevus Study (BTN), with assessment at age 12 (BTN1) and 14 (BTN2) [1]; the Memory, Attention, and Processing Speed Study (MAPS), with assessment at age 16 [2]; the 16UP study, with assessment at age 16-19 [3]; the Queensland Twin Imaging Study, with assessment at age 21-28 (QTIM) [4]; and the 19UP study of psychiatric symptoms, with assessment at age 18-36 [5]. All hair samples were collected between July 2012 and October 2015 in order to follow up the results of our pilot study [6]. The present cohort is independent of that used for the pilot study [6]. Zygosity was determined using standardized questions [7], and twin-pair photographs. In 201 of the 303 twin-pairs, zygosity was confirmed via genome-wide genotyping.

Hair sampling and HCC analysis

With the exception of the 16UP study, all hair samples were collected by a trained research assistant. In the 16UP study, hair samples were collected at home and sent by mail to the QIMR. In total, 821 samples were collected. HCC measurement was performed at the laboratory of two of the authors (M.B., T.B.). Four samples were discarded due to insufficient hair quantity (n=3) or shifted segmentation (n=1). Nine of the analyzed samples were <3 cm in length (7 with 2.5 cm; 2 with 2.0 cm). These did not differ from the 3 cm swatches in terms of average cortisol concentration (data not shown).

Cortisol concentration was measured as described by Binz et al. [8]. Briefly, after a 2-step-washing procedure (water 2 min, acetone 2 min), the dried hair samples were cut into snippets, spiked with the internal standard (2 ng D7-cortisone) and incubated in 5 mL MeOH at 55 °C overnight. The supernatant was evaporated under nitrogen at 35 °C and re-suspended in 150 µl MeOH and 350 µL reconstitution solution (2mM ammonium formate, 0.1 % formic acid). Cortisol was measured with a liquid chromatography-tandem mass spectrometry (LC-MS/MS) system in multiple reaction monitoring mode (MRM). The system consisted of a Shimadzu Prominence XR high pressure liquid-chromatography (HPLC) system coupled to a Sciex QTRAP® 5500 linear ion trap quadrupole mass spectrometer (Sciex, Darmstadt, Germany). All samples were centrifuged (5 min, 9000 rpm) in filter vials prior to LC-MS/MS analysis.

Influence of experimental covariates

The analysis demonstrated a decrease in HCC with increasing storage time. Additionally, significant mean differences were observed between the six studies from which the samples for the present analysis were drawn. Visual inspection of HCC according to month of collection revealed an approximate sinusoidal relationship with a maximum in March (at the end of Australian summer) and a minimum in September (at the end of Australian winter). Self and maternal ratings of the subjects' sun exposure showed a nominally significant ($p=0.03$) positive association. However, sun exposure was omitted from the model due to negligible improvement over including for monthly mean effects.

Statistical Analysis

Twin correlations, heritability of HCC, and shared covariance with psychological variables

Although the order in which variables are entered into a Cholesky decomposition does not affect the fit of the model, it does influence interpretation of the findings. The order of entry must therefore be determined according to the specific research question of interest. The present research question concerned: (i) the degree to which (genetic) variance in HCC is attributable to genes with an impact on the psychological variables stress, depression, and neuroticism; and (ii) the level of genetic variance for HCC remaining once these pleiotropic effects are removed. For the purposes of the present study, the key issue was to determine whether any of the three psychological variables had an impact, rather than to determine which of the three variables in particular was responsible for the effect. In view of this, all three psychological variables were entered into the model simultaneously.

Genotyping, quality control and imputation

The subset of 432 participants were genotyped in the context of a larger genome-wide association project that resulted in the genotyping of 28,028 individuals using the Illumina 317, 370, 610, 660, Core+Exome, PsychChip, Omni2.5 and OmniExpress SNP chips which included data from twins, their siblings and their parents. Genotype data were screened for genotyping quality (GenCall < 0.7), SNP and individual call rates (< 0.95), HWE failure ($P < 10^{-6}$) and MAF (< 0.01). As these samples were genotyped in the context of a larger project, the data were integrated with the larger QIMR genotype project and the data were checked for pedigree, sex and Mendelian errors and for non-European ancestry. As the QIMR genotyping

project included data from the multiple chip sets, to avoid introducing bias to the imputed data individuals genotyped on the Human Hap Illumina chips (the 317, 370, 610, 660K chips) were imputed separately from those genotyped on the Omni chips (the Core+Exome, PsychChip, Omni2.5 and OmniExpress chips). Individuals were imputed to the Haplotype Reference Consortium (HRC.1.1) [http://www.haplotype-reference-consortium.org/] [9] using a set of SNPs common to the first generation genotyping platforms (N ~ 278,000). Imputation was performed on the Michigan Imputation Server (https://imputationserver.sph.umich.edu/i) using the SHAPEIT/minimac Pipeline described at http://genome.sph.umich.edu/wiki/Minimac.

The PC account for population stratification—allele frequency differences between cases and controls due to systematic ancestry differences. PC were calculated using ‘smartpca’ as found in EigenSoft 6.0.1 (http://www.hsph.harvard.edu/alkes-price/software/). PCs were projected onto 20 PC axes and ancestry outliers (mean ± 6 SD in PC1, PC2 for the EUTWIN/HapMap European populations) were excluded.

Polygenic Risk Score Analysis

PLINK 1.90 (version 3, May 2016, https://www.cog-genomics.org/plink2/) was used to compute PRS for eight p-value thresholds (5e-8, 1e-5, 0.001, 0.01, 0.05, 0.1, 0.5, 1.0), in accordance with the procedure described by Wray et al. [10]. Although decreasing the significance threshold for inclusion of variants in the PRS increases the proportion of false positives contributing to the PRS, the aim of these analyses is to index the sum of all common variants influencing the trait and as such, it is important to obtain the most complete representation of the genetic architecture of the trait possible. To improve the accuracy of the PRS, the scores are built using

probabilistic (or dosage) genotypes to account for imputation uncertainty. As described in Wray et al [10], the variants available in both the discovery and target samples are selected and the variant with the highest test statistic in each linkage disequilibrium (LD) block is retained for the calculations. PRS are then estimated as the sum of risk alleles weighted by their respective independently estimated effect sizes. Only single nucleotide polymorphisms (SNPs) with a minor allele frequency of $\geqslant$ 0.01 and imputation quality of $r^2 \geqslant$ 0.6 were used in the calculation of the PRS. Genotypes were LD pruned using clumping in order to obtain SNPs in approximate LD (r^2 of 0.1 within a 10,000 base pair window). The PRS were standardized for further analysis.

To control for family structure, associations of PRS with HCC and the psychological variables were tested using linear mixed regression models in GCTA (Genome-wide Complex Trait Analysis v. 1.26) [11]. One-sided p-values are reported, according to the hypothesis of a positive association of the PRS with HCC and each of the psychological variables. P-values were calculated using the t-statistic and on the basis of the ß and standard error from the GCTA output. Variance explained by the PRS was calculated using: var(x) × b2/ var(y), where x is the PRS, b is the estimate of the fixed effect from GCTA, and y is the phenotype.

Supplementary text references

1 Zhu, G. *et al.* A genome-wide scan for naevus count: linkage to CDKN2A and to other chromosome regions. *European Journal of Human Genetics* **15**, 94-102 (2007).

2 Wright, M. *et al.* Genetics of cognition: outline of a collaborative twin study. *Twin Res* **4**, 48-56 (2001).

3 Kirk, K. M. *et al.* 16Up: Outline of a study investigating well-being and information and communication technology use in adolescent twins. (in prep.).

4 Blokland, G. A. *et al.* Quantifying the heritability of task-related brain activation and performance during the N-back working memory task: a twin fMRI study. *Biological psychology* **79**, 70-79 (2008).

5 Couvy-Duchesne, B. *et al.* Cohort Profile: The nineteen and up study, mapping neurobiological changes across mental health stages. (in press).

6 Rietschel, L. *et al.* Hair Cortisol and Its Association With Psychological Risk Factors for Psychiatric Disorders: A Pilot Study in Adolescent Twins. *Twin Res Hum Genet* **19**, 438-446, doi:10.1017/thg.2016.50 (2016).

7 Martin, N. G. & Martin, P. G. The inheritance of scholastric abilities in a sample of twins. I. Ascertainments of the sample and diagnosis of zygosity. *Ann Hum Genet* **39**, 213-218 (1975).

8 Binz, T. M., Braun, U., Baumgartner, M. R. & Kraemer, T. Development of an LC-MS/MS method for the determination of endogenous cortisol in hair using (13)C3-labeled cortisol as surrogate analyte. *J Chromatogr B Analyt Technol Biomed Life Sci* **1033-1034**, 65-72, doi:10.1016/j.jchromb.2016.07.041 (2016).

9 McCarthy, S. *et al.* A reference panel of 64,976 haplotypes for genotype imputation. *bioRxiv*, 035170 (2016).

10 Wray, N. R. & Maier, R. Genetic Basis of Complex Genetic Disease: The Contribution of Disease Heterogeneity to Missing Heritability. *Current Epidemiology Reports* **1**, 220-227, doi:10.1007/s40471-014-0023-3 (2014).

11 Yang, J., Lee, S. H., Goddard, M. E. & Visscher, P. M. Genome-wide complex trait analysis (GCTA): methods, data analyses, and interpretations. *Methods Mol Biol* **1019**, 215-236, doi:10.1007/978-1-62703-447-0_9 (2013).

Supplementary Tables

Supp. Table 1: Family structure

a) Overview of the final sample

Type	Family	Pair	Singleton	N
Singleton	11		11	11
TW-Sibling	2		4	4
Twin-pair	283	283	36	602
Triplet	14	14	14	42
2 Pair-Twins	3	6		12
Total	313	303	65	671

b) Overview of subjects assessed twice

Type	Family	Pair	Singleton	N
Twin-pair	67	67	4	138
Triplet	2	2		4
2 Pair-Twins	2	2		4
Total	71	71	4	146

c) Overview of subjects with genotype data

Type	Family	Pair	Singleton	N
Twin-pair	201	186	31	403
Triplet	7	7	7	21
2 Pair-Twins	2	4		8
Total	210	197	38	432

Supp. Table 2: Number of hair samples included in the final sample listed by study

Study	Time point 1	Time point 2	Study total
**BTN1**	343		343
**BTN2**	195	73#	268
**MAPS**	48		48
**QTIM**	34		34
**16UP**	48	73§	121
**19UP**	3		3
**Total**	671	146	817

73 subjects, who participated in the BTN1 study at time point 1, provided a second hair sample at time point 2 in the frame of the BTN2 study; § 73 subjects who participated in the BTN2 study at time point 1, provided a second hair sample at time point 2 in the frame of the 16UP study. BTN = Brisbane Twin Nevus Study; MAPS = Memory, Attention, and Processing Speed Study; QTIM = Queensland Twin Imaging Study

Supp. Table 3: Sum scores (SD) and n for the psychological questionnaires

Questionnaire	Total	Male	Female
DLSS*	25.05 (13.64) [n=632]	25.34 (14.32) [n=238]	24.87 (13.23) [n=394]
PSS*	17.01 (6.15) [n=132]	15.53 (5.40) [n=55]	18.06 (6.46) [n=77]
SPHERE	8.76 (6.93.) [n=616]	8.23 (7.09) [n=231]	9.08 (6.83) [n=385]
JEPQ	9.41 (5.05) [n=538]	8.86 (5.18) [n=197]	9.74 (4.95) [n=341]
NEO-FFI-R	23.18 (6.70) [n=82]	21.44 (7.18) [n=34]	24.42 (6.24) [n=48]

Abbreviations: SD=standard deviation, DLSS= Daily Life and Stressors Scale, PSS= Perceived Stress Scale, SPHERE= Somatic and Psychological Health Report, JEPQ= Junior Eysenck Personality Questionnaire, NEO-FFI-R= NEO-Five Factor Inventory revised version. *Both questionnaires have been used to create IRT scores for perceived stress

Supp. Table 4: Number of subjects of the cohorts used for calculation of the major depressive disorder polygenic risk score

**Dataset**	**N cases**	**N controls**
GERA	7,162	38,307
deCODE	1,980	9,536
Generation Scotland	997	6,358
iPSYCH	16,242	15,847
UK Biobank	8,248	16,089
PGC-MDD core	14,895	23,937
Total	49,524	110,074

GERA = Resource for Genetic Epidemiology Research on Adult Health and Aging; iPSYCH = The Lundbeck Foundation Initiative for Integrative Psychiatric Research; PGC-MDD = Psychiatric Genomics Consortium, Major Depressive Disorders working group

Supp. Table 5: Regression-coefficients (B) of sex and age effects of hair cortisol and the psychological variables

	Sex [a]	Age	Age2	Sex*Age	Sex*age^2
**Perceived stress [b]**	-.028	.057**	.000	-.134*	-.002
**Depressive symptoms [c]**	-.210$^{\#}$	.061*	-.067	-.093	.066
**Neuroticism [d]**	-.456**	.133**	-.232**	-.279*	.272*
**Hair cortisol [e]**	-.214**	.028	-.004	-.064	-.008

**p<0.01; *p<0.05; [#]p<0.1; [a]Sex was coded with 0=female and 1=males, as such negative B indicates higher values in female participants, [b]IRT-scores deriving from perceived stress questionnaires (DLS and PSS); [c]IRT-scores of the SPHERE [d]z-standardized values of the neuroticism questionnaires; [e]residualized and log-transformed values of hair cortisol

Supp. Table 6: Influence of covariates on HCC

									Variance change %		
**Model**	**Variance (95%CI)**		**-2LL**	**df**	**Compare**	**chi-sq.**	**Δdf**	***P***	**Full**	**BATCH only**	**No COVs**
FULL	0.7254	(0.6677-0.7899)	2740.8	1033					**-**	**14.6**	**25.5**
BATCH only	0.8495	(0.7819-0.9251)	2912.8	1053	2 &1	172.1	20	3.45e-26	**14.6**	**-**	**7.1**
No COVs	0.9102	(0.8378-0.9912)	2988.0	1087	3 & 2	75.2	34	6.06e-05	**20.3**	**6.7**	**-**
No BATCH	0.7707	(0.7094-0.8393)	2806.8	1067	4 & 1	66.1	34	8.04e-04	**5.9**	**10.2**	**18.1**
No STORAGE	0.7384	(0.6797-0.8041)	2760.2	1037	5 & 1	19.4	4	6.41e-04	**1.8**	**15.0**	**23.3**
No MONTH	0.7542	(0.6942-0.8213)	2783.3	1044	6 & 1	42.5	11	1.33e-05	**3.8**	**12.6**	**20.7**
No STUDY	0.7749	(0.7133-0.8439)	2812.8	1038	7 & 1	72.0	5	3.88e-14	**6.4**	**9.6**	**17.5**

Abbreviations: CI=confidence interval, -2LL=minus twice the log likelihood, df=degrees of freedom; p=p-value in chi-square, Δdf=change in degrees of freedom; no COVs=model including no covariates. HCC=hair cortisol concentration

Supp. Table 7: Phenotypic correlations (95% CI) between the psychological variables and HCC

	Perceived stress		Depressive symptoms		Neuroticism		Hair cortisol
Perceived stress	1						
Depressive symptoms	0.59	(0.54-0.64)	1				
Neuroticism	0.67	(0.63-0.72)	0.64	(0.56-0.69)	1		
Hair cortisol	0.04	(-0.04-0.12)	0.07	(-0.01-0.15)	0.08	(-0.00-0.16)	1

Abbreviations: CI=confidence interval. All calculations were corrected for sex, age, age^2, sex x age, sex x age^2.

Supp. Table 8: AE Model in the younger half of the sample for the psychological variables and HCC (n= 338; 210 females; age mean= 12.37±.54 years; median=12.24; range=10.1-14.01 years)

		Standardized Path Coefficients				Standardized Parameters %				Correlations			
		**STR**	**DEP**	**NEU**	**HCC**	**STR**	**DEP**	**NEU**	**HCC**	**STR**	**DEP**	**NEU**	**HCC**
Genetic (A)	STR	0.68				46.43				1			
	DEP	0.59	0.42			34.46	17.65			0.81	1		
	NEU	0.59	0.14	0.42		34.48	1.84	17.58		0.80	0.76	1	
	HCC	0.20	-0.06	0.16	0.82	3.96	0.39	2.51	67.19	0.23	0.15	0.28	1
Unshared Environment (E)	STR	0.73				53.57				1			
	DEP	0.26	0.65			5.51	42.38			0.34	1		
	NEU	0.39	0.22	0.51		15.52	4.71	25.87		0.58	0.50	1	
	HCC	-0.04	-0.07	-0.04	0.50	0.12	0.45	0.16	25.22	0.69	-0.10	-0.06	1

Abbreviations: STR=perceived Stress, DEP=depressive symptoms, NEU=neuroticism, HCC=hair cortisol concentration.

Supp. Table 9: AE Model in the older half of the sample for the psychological variables and HCC (n= 333; 209 females; age mean= 15.70 ± 2.32 years; median=14.57; range=14.02-31.1 years)

		Standardized Path Coefficients				Standardized Parameters %				Correlations			
		**STR**	**DEP**	**NEU**	**HCC**	**STR**	**DEP**	**NEU**	**HCC**	**STR**	**DEP**	**NEU**	**HCC**
Genetic (A)	STR	0.81				66.16				1			
	DEP	0.54	0.54			29.28	29.33			0.71	1		
	NEU	0.58	0.18	0.49		33.32	3.10	23.68		0.75	0.69	1	
	HCC	0.03	0.10	0.03	0.82	0.08	1.02	0.06	67.58	0.04	0.11	0.07	1
Unshared Environment (E)	STR	0.58				33.84				1			
	DEP	0.32	0.56			10.47	30.92			0.50	1		
	NEU	0.27	0.27	0.51		7.15	7.05	25.70		0.42	0.58	1	
	HCC	-0.16	0.05	-0.07	0.53	2.52	0.26	0.42	28.06	0.28	0.22	0.07	1

Abbreviations: STR=perceived Stress, DEP=depressive symptoms, NEU=neuroticism, HCC=hair cortisol concentration.

Supp. Table 10: HCC predicted by plasma cortisol PRS

p-value threshold	Beta	SE	t	p 1-sided+	p 2-sided	R^2 %
**<5e-8**	-0.09281	0.04077	-2.27633	0.98834	0.02333	1.48%
**<1e-5**	-0.05341	0.04427	-1.20654	0.88586	0.22829	0.40%
**<0.001**	-0.06876	0.03943	-1.74382	0.95904	0.08192	0.84%
**<0.01**	0.01534	0.04156	0.36919	0.35608	0.71217	0.04%
**<0.05**	-0.00018	0.04085	-0.00448	0.50179	0.99642	0.00%
**<0.1**	0.01609	0.04165	0.38643	0.34969	0.69938	0.04%
**<0.5**	0.02568	0.04266	0.60205	0.27373	0.54746	0.10%
**<1.0**	0.02542	0.04294	0.59183	0.27714	0.55428	0.10%

Abbreviations: PRS=polygenic risk score, HCC=hair cortisol concentration, SE=standard error

Supp. Table 11: HCC predicted by MDD PRS

p-value threshold	Beta	SE	t	p 1-sided+	p 2-sided	R^2 %
**<5e-8**	-0.03709	0.04497	-0.82475	0.79501	0.40998	0.21%
**<1e-5**	0.00961	0.04247	0.22623	0.41056	0.82113	0.02%
**<0.001**	0.01353	0.04358	0.31045	0.37819	0.75637	0.03%
**<0.01**	0.02184	0.04169	0.52385	0.30033	0.60066	0.08%
**<0.05**	0.02511	0.04348	0.57758	0.28193	0.56386	0.10%
**<0.1**	0.01503	0.04035	0.37241	0.35489	0.70977	0.04%
**<0.5**	0.00633	0.04035	0.15695	0.43768	0.87536	0.01%
**<1.0**	0.00810	0.04065	0.19936	0.42104	0.84208	0.01%

Abbreviations: PRS=polygenic risk score, HCC=hair cortisol concentration, MDD=major depressive disorder, SE=standard error

Supp. Table 12: HCC predicted by neuroticism PRS

p-value threshold	Beta	SE	t	p 1-sided+	p 2-sided	R^2 %
**<5e-8**	-0.04756	0.04479	-1.06173	0.85551	0.28897	0.35%
**<1e-5**	-0.04728	0.04651	-1.01666	0.84505	0.30990	0.33%
**<0.001**	-0.01165	0.04211	-0.27672	0.60894	0.78213	0.02%
**<0.01**	-0.02199	0.04277	-0.51411	0.69628	0.60745	0.08%
**<0.05**	-0.00087	0.04347	-0.02002	0.50798	0.98404	0.00%
**<0.1**	0.00548	0.04248	0.12904	0.44869	0.89739	0.00%
**<0.5**	0.01033	0.04236	0.24385	0.40373	0.80747	0.02%
**<1.0**	0.01082	0.04213	0.25691	0.39869	0.79737	0.02%

Abbreviations: PRS=polygenic risk score, HCC=hair cortisol concentration, SE=standard error

Supp. Table 13: Perceived stress predicted by plasma cortisol PRS

p-value threshold	Beta	SE	t	p 1-sided+	p 2-sided	R^2 %
**<5e-8**	-0.00309	0.03051	-0.10112	0.54025	0.91950	0.00%
**<1e-5**	-0.00779	0.03306	-0.23570	0.59311	0.81378	0.02%
**<0.001**	-0.01108	0.02948	-0.37586	0.64640	0.70721	0.04%
**<0.01**	-0.00114	0.03093	-0.03681	0.51467	0.97065	0.00%
**<0.05**	0.01070	0.03038	0.35232	0.36239	0.72477	0.03%
**<0.1**	-0.01468	0.03095	-0.47437	0.68226	0.63549	0.06%
**<0.5**	0.00887	0.03174	0.27945	0.39002	0.78004	0.02%
**<1.0**	0.01116	0.03193	0.34947	0.36346	0.72691	0.03%

Abbreviations: PRS=polygenic risk score, SE=standard error

Supp. Table 14: Perceived stress predicted by MDD PRS

p-value threshold	Beta	SE	t	p 1-sided+	p 2-sided	R^2 %
**<5e-8**	0.07122	0.03287	2.16676	0.01541	0.03082	1.36%
**<1e-5**	0.01778	0.03134	0.56731	0.28541	0.57081	0.10%
**<0.001**	0.02576	0.03223	0.79916	0.21232	0.42465	0.18%
**<0.01**	0.05560	0.03073	1.80944	0.03555	0.07110	0.92%
**<0.05**	0.03516	0.03222	1.09108	0.13793	0.27587	0.33%
**<0.1**	0.04070	0.02981	1.36528	0.08645	0.17290	0.53%
**<0.5**	0.01800	0.02995	0.60096	0.27410	0.54819	0.10%
**<1.0**	0.02298	0.03015	0.76232	0.22315	0.44630	0.16%

Abbreviations: PRS=polygenic risk score, MDD=major depressive disorder, SE=standard error

Supp. Table 15: Perceived stress predicted by neuroticism PRS

p-value threshold	Beta	SE	t	p 1-sided+	p 2-sided	R^2 %
**<5e-8**	-0.03817	0.03341	-1.14245	0.87304	0.25392	0.40%
**<1e-5**	-0.02442	0.03448	-0.70834	0.76043	0.47913	0.16%
**<0.001**	-0.01135	0.03125	-0.36328	0.64171	0.71658	0.04%
**<0.01**	0.00136	0.03182	0.04260	0.48302	0.96604	0.00%
**<0.05**	0.02517	0.03227	0.77984	0.21796	0.43593	0.17%
**<0.1**	0.02466	0.03151	0.78274	0.21711	0.43422	0.17%
**<0.5**	0.03031	0.03130	0.96860	0.16665	0.33331	0.27%
**<1.0**	0.03057	0.03113	0.98186	0.16337	0.32674	0.28%

Abbreviations: PRS=polygenic risk score, SE=standard error

Supp. Table 16: Depressive symptoms predicted by plasma cortisol PRS

p-value threshold	Beta	SE	t	p 1-sided+	p 2-sided	R^2 %
**<5e-8**	0.00095	0.05383	0.01759	0.49299	0.98598	0.00%
**<1e-5**	-0.05429	0.05628	-0.96468	0.83233	0.33534	0.30%
**<0.001**	-0.00751	0.05079	-0.14786	0.55873	0.88253	0.01%
**<0.01**	0.02289	0.05236	0.43722	0.33111	0.66221	0.06%
**<0.05**	0.01663	0.05186	0.32071	0.37431	0.74861	0.03%
**<0.1**	-0.01843	0.05376	-0.34288	0.63406	0.73189	0.04%
**<0.5**	-0.01933	0.05449	-0.35470	0.63849	0.72302	0.04%
**<1.0**	-0.01924	0.05475	-0.35132	0.63723	0.72555	0.04%

Abbreviations: PRS=polygenic risk score, SE=standard error

Supp. Table 17: Depressive symptoms predicted by MDD PRS

p-value threshold	Beta	SE	t	p 1-sided+	p 2-sided	R^2 %
**<5e-8**	0.15033	0.05740	2.61887	0.00459	0.00919	2.34%
**<1e-5**	0.07630	0.05450	1.39985	0.08120	0.16240	0.68%
**<0.001**	0.08800	0.05504	1.59887	0.05535	0.11071	0.87%
**<0.01**	0.08382	0.05214	1.60765	0.05438	0.10877	0.85%
**<0.05**	0.07601	0.05582	1.36181	0.08705	0.17409	0.62%
**<0.1**	0.08865	0.05151	1.72107	0.04304	0.08608	1.00%
**<0.5**	0.06665	0.05218	1.27735	0.10114	0.20229	0.54%
**<1.0**	0.06823	0.05256	1.29817	0.09752	0.19504	0.56%

Abbreviations: PRS=polygenic risk score, MDD=major depressive disorder, SE=standard error

Supp. Table 18: Depressive symptoms predicted by neuroticism PRS

p-value threshold	Beta	SE	t	p 1-sided+	p 2-sided	R^2 %
**<5e-8**	-0.02833	0.05845	-0.48465	0.68589	0.62821	0.08%
**<1e-5**	0.01849	0.05961	0.31015	0.37831	0.75662	0.03%
**<0.001**	0.04982	0.05531	0.90084	0.18413	0.36826	0.27%
**<0.01**	0.05962	0.05537	1.07678	0.14114	0.28229	0.37%
**<0.05**	0.10135	0.05623	1.80233	0.03616	0.07231	1.04%
**<0.1**	0.10096	0.05531	1.82544	0.03437	0.06874	1.08%
**<0.5**	0.08785	0.05451	1.61154	0.05396	0.10792	0.89%
**<1.0**	0.08753	0.05432	1.61125	0.05399	0.10798	0.89%

Abbreviations: PRS=polygenic risk score, SE=standard error

Supp. Table 19: Neuroticism predicted by plasma cortisol PRS

p-value threshold	Beta	SE	t	p 1-sided+	p 2-sided	R^2 %
**<5e-8**	-0.00710	0.05317	-0.13345	0.55304	0.89391	0.01%
**<1e-5**	0.04342	0.05595	0.77594	0.21914	0.43828	0.18%
**<0.001**	0.01148	0.05031	0.22821	0.40981	0.81961	0.02%
**<0.01**	0.00212	0.05185	0.04083	0.48373	0.96745	0.00%
**<0.05**	0.02833	0.05127	0.55247	0.29048	0.58096	0.09%
**<0.1**	0.01324	0.05323	0.24867	0.40188	0.80376	0.02%
**<0.5**	0.04961	0.05394	0.91978	0.17915	0.35829	0.25%
**<1.0**	0.04878	0.05418	0.90026	0.18428	0.36857	0.24%

Abbreviations: PRS=polygenic risk score, SE=standard error

Supp. Table 20: Neuroticism predicted by MDD PRS

p-value threshold	Beta	SE	t	p 1-sided+	p 2-sided	R^2 %
**<5e-8**	0.11231	0.05671	1.98055	0.02419	0.04839	1.21%
**<1e-5**	-0.01330	0.05393	-0.24658	0.59731	0.80537	0.02%
**<0.001**	0.05856	0.05448	1.07481	0.14158	0.28316	0.36%
**<0.01**	0.04526	0.05167	0.87603	0.19079	0.38158	0.23%
**<0.05**	0.05997	0.05529	1.08479	0.13936	0.27872	0.36%
**<0.1**	0.05466	0.05098	1.07225	0.14216	0.28431	0.35%
**<0.5**	0.04277	0.05166	0.82792	0.20413	0.40825	0.20%
**<1.0**	0.04244	0.05204	0.81542	0.20768	0.41536	0.20%

Abbreviations: PRS=polygenic risk score, MDD=major depressive disorder, SE=standard error

Supp. Table 21: Neuroticism predicted by neuroticism PRS

p-value threshold	Beta	SE	t	p 1-sided+	p 2-sided	R^2 %
**<5e-8**	-0.06115	0.05778	-1.05832	0.85470	0.29060	0.36%
**<1e-5**	-0.07206	0.05881	-1.22546	0.88941	0.22119	0.47%
**<0.001**	-0.03929	0.05477	-0.71726	0.76317	0.47367	0.16%
**<0.01**	-0.00935	0.05497	-0.17009	0.56748	0.86504	0.01%
**<0.05**	0.00827	0.05603	0.14758	0.44138	0.88276	0.01%
**<0.1**	0.00880	0.05513	0.15969	0.43661	0.87321	0.01%
**<0.5**	0.01311	0.05402	0.24274	0.40417	0.80834	0.02%
**<1.0**	0.01744	0.05383	0.32408	0.37303	0.74606	0.03%

Abbreviations: PRS=polygenic risk score, SE=standard error
